# Microglia: A Potential Therapeutic Target for Sepsis-Associated Encephalopathy and Sepsis-Associated Chronic Pain

**DOI:** 10.3389/fphar.2020.600421

**Published:** 2020-11-27

**Authors:** Yi Li, Lu Yin, Zhongmin Fan, Binxiao Su, Yu Chen, Yan Ma, Ya Zhong, Wugang Hou, Zongping Fang, Xijing Zhang

**Affiliations:** Department of Anaesthesiology and Perioperative Medicine, Xijing Hospital, The Fourth Military Medical University, Xi’an, China

**Keywords:** sepsis, sepsis-associated encephalopathy, chronic pain, microglia, neuroinflammation

## Abstract

Neurological dysfunction, one of the severe manifestations of sepsis in patients, is closely related to increased mortality and long-term complications in intensive care units, including sepsis-associated encephalopathy (SAE) and chronic pain. The underlying mechanisms of these sepsis-induced neurological dysfunctions are elusive. However, it has been well established that microglia, the dominant resident immune cell in the central nervous system, play essential roles in the initiation and development of SAE and chronic pain. Microglia can be activated by inflammatory mediators, adjacent cells and neurotransmitters in the acute phase of sepsis and then induce neuronal dysfunction in the brain. With the spotlight focused on the relationship between microglia and sepsis, a deeper understanding of microglia in SAE and chronic pain can be achieved. More importantly, clarifying the mechanisms of sepsis-associated signaling pathways in microglia would shed new light on treatment strategies for SAE and chronic pain.

## Introduction

Sepsis is a primary fatal syndrome in intensive care units (ICUs) that is characterized by an imbalance in the pro- and anti-inflammatory systems after infection (van der Poll et al., 2017). Sepsis is commonly accompanied by multiorgan dysfunctions and causes severe disturbances in the nervous system, such as sepsis-associated encephalopathy (SAE) and chronic pain (Shankar-Hari and Rubenfeld, 2016). SAE is defined as diffuse cerebral dysfunction during sepsis, while sepsis-associated chronic pain is partly a reflection of central sensitization with synaptic plasticity and increased neuronal responsiveness (Helbing et al., 2018; Ji et al., 2018). Both of these conditions can lead to long-lasting decrements in health-related quality of life for patients. To be specific, it has been demonstrated that SAE can cause long-term neurological deficits, including cognitive deficits (10–20%) and anxiety and stress disorders (10–30%) (Iwashyna et al., 2010; Battle et al., 2013; Helbing et al., 2018). Moreover, 31–70% of sepsis survivors claim to suffer from pain 6 months after discharge from the hospital (Carpenter et al., 2019). Given that there is still a lack of specific treatment options proposed for SAE and sepsis-associated chronic pain, understanding the mechanisms of the pathological alterations in the brain is essential for developing effective treatments to avoid the potentially devasting effects of sepsis.

Microglia, the resident immune cells in the brain, modulate multiple brain functions via cytokines and their interactions with neurons and nonneuronal cells (Greenhalgh et al., 2020). Cytokines protect the immune system against adverse stimuli and maintain homeostasis, but cytokine dysregulation can induce neurotoxicity, as has been verified in patients with neurodegenerative disorders (van Gool et al., 2010). The relationship between systemic infection and microglial activation in the brain was confirmed in septic patients more than a decade ago (Lemstra et al., 2007). Microglial activation in the brain was further demonstrated in an animal model of sepsis induced by lipopolysaccharide (LPS) and caecal ligation and puncture (Szollosi et al., 2018). SAE and sepsis-associated chronic pain are thought to be the consequences of inflammation in the brain (Michels et al., 2015a; Baumbach et al., 2016). Moreover, microglial activation contributes to neuroinflammation during sepsis; thus, it is believed that microglial activation plays an essential role in SAE and sepsis-associated chronic pain (Sharshar et al., 2014). The correlation between activated microglia and neurological deficits in sepsis has been reported in a few studies; however, the underlying mechanisms need to be further explored.

**FIGURE 1:**
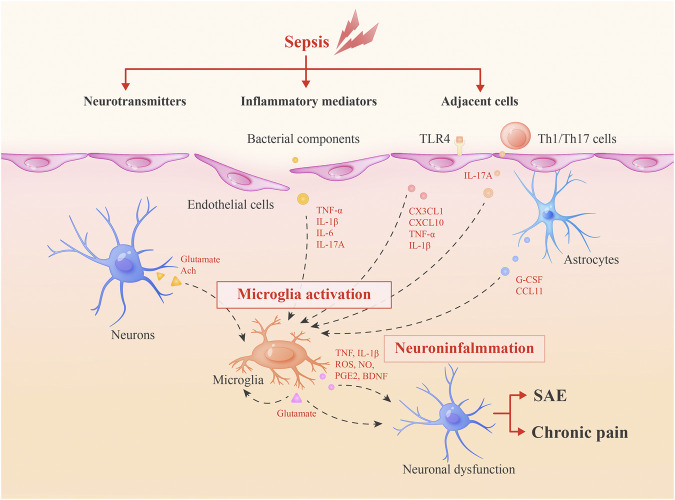
Roles of microglia in SAE and sepsis-associated chronic pain. During sepsis, microglia can be activated by inflammatory mediators, adjacent cells and neurotransmitters. Then, activated microglia integrate neuroinflammation and cause neuronal dysfunctions via secreted inflammatory mediators or glutamate to lead to SAE and chronic pain. Glutamate may form a positive feedback mechanism in microglia activation.

In this review, we summarize the current knowledge of the activation of microglia during sepsis and roles of these cells in long-term cognitive deficits and chronic pain, to shed new light on the development of novel therapeutics for SAE and sepsis-associated chronic pain (Figure 1).

### Activation of Microglia in the Central Nervous System During Sepsis

Under physiological conditions, microglia are in a resting state, characterized by branched morphology and surveillance of the microenvironment to maintain homeostasis; when these cells encounter pathological insults, this resting phenotype will switch to an activated amoeboid form and exert destructive or neuroprotective effects (Hoogland et al., 2015). Microglial activation in the brain has been observed in both LPS-treated murine models and patients who died from sepsis, suggesting that microglia are relevant to systemic inflammation and pathology in the central nervous system (CNS) (Fukushima et al., 2015; Westhoff et al., 2019). Peripheral inflammation induces the immune response in the CNS via several pathways, which might contribute to the considerable roles of microglia in sepsis.

#### 1. Inflammatory Mediators

Bacterial components stimulate microglia via pattern recognition receptors such as Toll-like receptors (TLR-2, TLR-4, and TLR-9) and nucleotide-binding oligomerization domain-2 (NOD2) (Ye et al., 2019). In addition, it has been verified that cytokines (particularly tumour necrosis factor α (TNF-α), interleukin 1β (IL-1β), and interleukin 6 (IL-6)), which are generated peripherally during sepsis, transmit signals through the impaired blood-brain barrier (BBB) and activate microglia to modulate neuronal function (van Gool et al., 2010). The IL-17A/IL-17R signaling pathway has also been reported to play an important role in stimulating microglia. The IL-17A/IL-17R signaling pathway facilitates microglial secretion of inflammatory factors, including IL-1β and IL-23, which exacerbate the secretion of IL-17A from immune cells and create a vicious cycle of sustained, amplified inflammation in the brain (Ye et al., 2019).

#### 2. Adjacent Cells

Adjacent cells have modulatory effects on microglia. Astrocytic end-feet express multiple cytokine receptors and attach to vascular endothelial cells or leptomeningeal cells in the peripheral blood vessels, allowing astrocytes to initially respond to systemic inflammation (Shimada and Hasegawa-Ishii, 2017). Then, inflammatory signals are conveyed and activate microglia through astrocyte-generated cytokines, such as granulocyte-colony stimulating factor (G-CSF) and CCL11(Shimada and Hasegawa-Ishii, 2017). G-CSF acts as a microglial growth factor, and CCL11 can promote the microglial migration to inflamed foci and facilitate microglial production of reactive oxygen species (ROS), ultimately resulting in excitotoxic neuronal death (Shimada and Hasegawa-Ishii, 2017). Simultaneously, in cultured cells, astrocytes produce anti-inflammatory substances, including transforming growth factor (TGF) and prostaglandin E2 (PGE2), which downregulate microglial activity and restrict neuroinflammation (Michels et al., 2017). However, whether this anti-inflammatory effect exists *in vivo* of murine models of sepsis has not been fully studied.

Endothelial cells also play important roles in modulating microglial functions. Wang, H. et al. showed that endothelial cell activation can result in increased leukocyte adhesion, which is responsible for elevated CX3CL1 expression on endothelial cells, activating and guiding microglia toward the inflamed brain during sepsis (Wang et al., 2015). Furthermore, TLR-4 on endothelial cells is also an important inducer of microglial activation by regulating the secreting of CXCL10, TNF-α and IL-1β(Chen and Trapp, 2016). In the experimental autoimmune encephalomyelitis (EAE) mouse model, it was demonstrated that peripheral Th1/Th17 cells were initially recruited to the brain and produced massive amounts of IL-17A, inducing the activation of resident microglia and prolonging the inflammatory response (Murphy et al., 2010). Since the IL-17A/IL-17R pathway is a vital component in microglia-mediated neuroinflammation in sepsis, it is possible that Th1/Th17 cells play a similar role in this context.

#### 3. Neurotransmitters

Microglia express receptors of various neurotransmitters, including glutamate, acetylcholine (Ach), and so on, which cooperate with each other to maintain normal neuronal functions (Wolf et al., 2017). During sepsis, imbalanced expressions of these neurotransmitters exert negative effects on microglia and alter the equilibrium in the brain. In BV2 cells (a murine microglial cell line), LPS increases the Ca^2+^ concentration and upregulates CaMKII phosphorylation, thus inducing ERK1/2 and NF-κB phosphorylation, which stimulate microglial activation and the expression of pro-inflammatory cytokines and enzymes (Wu et al., 2018). These processes are regulated by overexpressed glutamate during sepsis and the inhibition of glutamate receptors (N-methyl-d-aspartate (NMDA) receptors) could attenuate those effects (Wu et al., 2018). On the contrary, Ach and nicotine can reduce LPS-induced TNF-α release from microglia through the activation of α-nAChR, which was reported to deliver an anti-inflammatory signal. However, the downregulation of cholinergic neurons during sepsis eliminates this effect (Dal-Pizzol et al., 2014).

## Microglia in Sepsis-Associated Encephalopathy

### The Mechanisms of Sepsis-Associated Encephalopathy

SAE is a common but severe complication of sepsis. During SAE, the brain exhibits acute and long-lasting pathological alterations in mouse models and human patients. Cerebrovascular impairment and neuroinflammation are defined as the two main triggering mechanisms of SAE. On the one hand, impaired cerebrovascular-related changes in cerebral perfusion were observed in patients with severe sepsis or septic shock, which have been demonstrated to facilitate SAE (Schramm et al., 2012). On the other hand, brain cytokines and chemokines are generated within a few hours of sepsis onset in murine models, which lead to increased permeability of the BBB, causing immune cells, inflammatory factors and other substances in the periphery to enter the brain and induce disturbances (Comim et al., 2011). Cerebral endothelial activation is also a pathophysiological condition in the progression of SAE and has been reported to induce robust inflammation, abnormal BBB permeability, and even facilitate coagulation by the release of pro-inflammatory cytokines and nitric oxide (NO) (Sharshar et al., 2010; Heming et al., 2017).

Many other mechanisms are also likely to participate in the pathogenesis of SAE. Neuronal necrosis and apoptosis are thought to directly induce neuronal loss in the brain following LPS-induced SAE (Deng et al., 2013). And, it is possible that mitochondrial dysfunction and increased levels of reactive oxygen/nitrogen species can promote neuronal death (Heming et al., 2017). Astrocytes can be activated and exert effects during SAE via astrogliosis (Deng et al., 2013). In addition, activated microglia in this situation release inflammatory mediators to consequently impair the BBB and suppress neuronal activity (Deng et al., 2013). Complement cascade is also a phenomenon caused by overactive inflammation during sepsis, leading to deleterious effects in SAE (Jacob et al., 2007). Furthermore, changes in neurotransmission, such as glutamate-related neuronal excitotoxicity, facilitate neuronal apoptosis under the septic condition (Heming et al., 2017). The dopaminergic, β-adrenergic, cholinergic, and GABA receptor systems are all impaired during sepsis, which might result from NO produced by endothelial cells or elevated levels of circulating amino acids (Heming et al., 2017).

### Activated Microglia in SAE and Cognitive Dysfunction

Cognitive dysfunction is a common but severe consequence of SAE. Delirium is a typical symptom in the acute phase of SAE that occurs in 50% of patients (Mazeraud et al., 2020). Even after effective treatment, a large number of individuals who survive sepsis also develop long-term cognitive deficits and seem to have a higher risk of suffering from dementia (Widmann and Heneka, 2014). The correlation between those long-term brain dysfunctions and delirium in the acute phase of SAE has been reported (MacLullich et al., 2009), and the shared mechanisms underlying these two phenomena are receiving much more attention. Activated microglia have recently been proposed to play a role in accelerating SAE and have a close relationship with cognitive changes. Consistently, inhibition of microglia by an intracerebroventricular (ICV) injection of minocycline decreased acute brain oxidative damage, inflammation, and long-term cognitive impairment in sepsis survivors (Michels et al., 2015b). Thus, exploring the roles of microglia in SAE and long-term cognitive impairment will deepen our understanding of pathology of SAE and probably offer a potential therapeutic target for those patients.

Activated microglia lead to neuronal dysfunctions and memory impairment via releasing large amounts of pro-inflammatory mediators and inducing the expression of multiple enzymes during sepsis (Wu et al., 2018). For example, working memory, as well as hippocampal-dependent memory, has a negative correlation with the level of IL-1β, which can be released by activated microglia in SAE (Tang et al., 2018). In addition, Nox2 (a member of the NADPH oxidase family), GRK2 (G protein-coupled receptor kinase 2) and NO-producing enzymes (such as inducible nitric oxide synthase) in microglia regulate oxidative and nitrosative stress during inflammation, contributing to the amplification of pro-inflammatory mediators production, as well as long-term brain deficits after sepsis (Hernandes et al., 2014; Widmann and Heneka, 2014; Kawakami et al., 2018). Neurotransmitters can also be regulated by activated microglia. A moderate level of glutamate is released by neurons, astrocytes, and homeostatic microglia. However, toxic amounts of glutamate are produced by activated microglia through a mechanism that involves connexin channels and the cystine/glutamate antiporter system (Bozza et al., 2013; Michels et al., 2017). As glutamate has a regulatory effect on microglia, it seems that glutamate forms a positive feedback mechanism in microglia activation.

It is important to note that elderly individuals with neurodegenerative diseases have increased levels of innate inflammation in the brain and are vulnerable to delirium (van Gool et al., 2010). In animal sepsis models, neurobehavioural impairments are highly distinct in those with certain pre-existing conditions (Lemstra et al., 2007). Microglia are known to play crucial roles in mediating inflammatory processes associated with various neurodegenerative diseases (Tang et al., 2018). In these contexts, once microglia are activated or primed, this status is believed to be sustained for a long time (Lemstra et al., 2007). Thus, during sepsis, it is well accepted that when activated/primed microglia encounter a subsequent exogenous stimulus, these cells are inclined to exert an exaggerated response and produce enormous levels of cytokines, facilitating brain disturbances and exacerbating SAE (Lemstra et al., 2007).

## Microglia in Sepsis-Associated Chronic Pain

### The Mechanisms of Chronic Pain

Pain is a key defense system that enables the host to detect and avoid harmful stimuli. A complex and properly functioning neural circuit is necessary for the perception of pain. Briefly, noxious stimuli excite the peripheral ends of primary afferent sensory neurons, through which excitatory information is conveyed to the nociceptive projection neurons in the spinal dorsal horn and further up to those in the brainstem and higher brain regions, processing the sensory and affective components of pain (Inoue and Tsuda, 2018). Although acute pain is considered to exert a protective effect on the host, once the pain persists for more than 3 months and develops into chronic and recurrent pain, it becomes a disease condition (Milligan and Watkins, 2009). There are several types of chronic pain, including neuropathic pain, inflammatory pain, cancer-associated pain and drug-induced pain after chemotherapy and chronic opioid exposure. All of these types of pain are characterized by spontaneous pain, as well as evoked pain in response to noxious (hyperalgesia) or non-noxious (allodynia) stimuli (Ji et al., 2018).

Abnormal excitability in the CNS and the activation of glial cells, including astrocytes, microglia, oligodendrocytes in the CNS and satellite glial cells, Schwann cells in the peripheral nervous system, are believed to be the two basic pathogeneses contributing to chronic pain (Ji et al., 2013). Persistent peripheral inflammation leads to the increased release of neurotransmitters from the primary afferent central terminals to the spinal cord and brain, producing a state of neuronal hyperactivity and hyperexcitability (Ji et al., 2018). Besides, synaptic plasticity, LTP and disinhibition of inhibitory signaling can all contribute to pain hypersensitivity (Kyranou and Puntillo, 2012; Ji et al., 2018). Glia activation is another driver of chronic pain. These cells are regulated by several neuromodulators such as ATP, CSF, chemokines, neuropeptides and facilitate neuroinflammation and chronic pain via glia-produced factors indispensably (Ji et al., 2018). Notably, more and more researchers are proposing that neuroinflammation is highly effective in modulating pain sensitivity and is recognized to be associated with the persistence and chronification of human pain conditions (Ji et al., 2018). For example, TNF, IL-1β, and IL-6 rapidly modulate the function of neurotransmitter receptors to result in enhanced excitatory synaptic transmission and suppressed inhibitory synaptic transmission in the spinal pain circuit (Kawasaki et al., 2008). What’s more, neuroinflammation is also participating in inducing and sustaining spinal cord LTP in chronic pain (Ji et al., 2018).

### Activated Microglia in Sepsis-Associated Chronic Pain

Chronic pain is a frequently reported consequence of critical care illnesses, with 31–70% of septic patients claiming pain 6 months after discharge from the hospital (Carpenter et al., 2019). The most likely cause of this pain can be inflammation, and microglia are believed to contribute to this pathology. Microglia activation was observed in animal model of inflammatory pain as long ago as 1999 (Chen et al., 2018). And, functional imaging reveals glia activation in patients with chronic pain (Loggia et al., 2015).

Increasing evidence suggests that microglia drive central sensitization via microglial mediators, mainly TNF and IL-1β(Chen et al., 2018). For example, TNF induces spontaneous excitatory postsynaptic current by modulating the activities of TRPV1, AMPAR and NMDAR in spinal neurons (Chen et al., 2018). In addition, the cytokines PGE2 and brain-derived neurotrophic factor (BDNF) released by microglia can modulate inhibitory synaptic transmission via pre-, post- and extra-synaptic mechanisms. Disinhibition and LTP in the CNS also have tight correlations with activated microglia, as it has been suggested that TNF and IL-1β secreted by microglia are individual factors to induce LTP in spinal dorsal horn neurons (Ji et al., 2013; Chen et al., 2018). Intriguingly, microglia signaling is sex-dependent and spinal microglia play little or no role in regulating neuropathic pain in female rodents. This may result from the distinct expression levels of testosterone in male and female rodents and T cells appear to replace the role of microglia in neuropathic pain in female rodents (Sorge et al., 2015). Furthermore, spinal microglia priming also increases vulnerability to pain enhancement in mice via augmented microglia production of pro-inflammatory products (Hains et al., 2010).

Bacterial infections can induce pain in rodents. For example, the bacterium *staphylococcus aureus* causes pain not only in a direct manner through activating nociceptors, but also in an indirect manner via the intermediating effects of immune cells and secreted inflammatory substances (Chiu et al., 2013). What’s more, LPS has been demonstrated to produce pain hypersensitivity by sensitizing TRPV1 in nociceptors (Diogenes et al., 2011). Although an increasing number of studies are focusing on the roles of microglia in chronic pain as described above, it is worth noting that most studies are conducted after nerve injury, not bacterial infections in murine models. This can partly attribute to the absence of models mimicking and criteria evaluating the chronic pain after bacterial infections, particularly sepsis. For instance, after subcutaneous injection of bacteria in the mouse hid-paw, the infection-induced mechanical hypersensitivity seems to begin declining in 6 h and disappear in 72 h (Chiu et al., 2013). And it has been reported that shoulder pain is the most frequently reported chronic pain of ICU survivors, which is difficult to evaluate in murine models (Battle et al., 2013). The states and functions of microglia are distinct in different circumstances, such as in neuropathic pain and inflammatory pain (Milligan and Watkins, 2009). Therefore, there is still a long way to go in understanding the exact roles of microglia in sepsis and sepsis-associated chronic pain.

## Treatment

### Treatment of SAE

In recent years, our understanding of the pathophysiology of sepsis has improved. However, there is still no targeted treatment for sepsis or its complications: SAE and chronic pain. Strategies for SAE treatment are still based on the management of sepsis and the clinical manifestations of SAE, including seizure, delirium and coma (Mazeraud et al., 2020). The use of sedation is a controversial issue. Dexmedetomidine, an alpha agonist agent, has shown beneficial effects in patients with septic shock (Mazeraud et al., 2020). However, in a recent larger study, no significant advantage was observed for critically ill patients in the dexmedetomidine group (Shehabi et al., 2019). Moreover, benzodiazepines and opioids should be avoided since they are independent risk factors for the development of acute SAE in the ICU. Neuroprotective agents have also been considered options for SAE treatment. For example, impaired cholinergic neurotransmission has an important role in the development of delirium in SAE. However, rivastigmine, an inhibitor of acetylcholinesterase and butyrylcholinesterase, caused higher mortality and longer median duration of delirium than the placebo in critically ill patients (van Eijk et al., 2010). So, it is necessary to seek for new therapeutic strategies of SAE. For example, during SAE, synaptic activity is significantly reduced. Since GABA-A receptors participate in most neuronal inhibitory synapses, GABA-A may become a new target for the prevention and treatment of delirium (Molnar et al., 2018).

### Treatment of Chronic Pain

There is limited knowledge about sepsis-induced chronic pain. Experimental and clinical data show that there is a close relationship between inflammation and pain perception. As the most serious systemic inflammation, sepsis may induce not only acute changes in nociception but also long-lasting alterations in the CNS, increasing the risk of chronic pain conditions associated with ICU stays (Baumbach et al., 2016). For the treatment of pain, the first step includes non-opioid analgesics such as paracetamol and non-steroidal anti-inflammatory drugs (NSAIDs), which reduce inflammation and pain by reducing the effects of the cyclooxygenases COX-1 and COX-2 and reduce the production of inflammatory mediators. In the second step of pain treatment, the weak opioids codeine and dihydrocodeine are added. Opioids mimic the effects of natural pain by reducing the chemicals (endorphins) that activate opioid receptors in the CNS, thereby reducing the transmission of noxious signals. The third step requires strong opioids, which are more effective than weak opioids but also have more serious side effects (Hylands-White et al., 2017). Other treatment approaches for chronic pain management includes: adjuvant drugs, dealing with cognitive, emotional and behavioural disorders in chronic pain conditions, psychological and social approaches (Hylands-White et al., 2017). Besides, with advances in our understanding of mechanisms of chronic pain, cell-based therapy is now an option for treating chronic pain, such as stem cell therapy and so on (Chakravarthy et al., 2017).

### Microglia as the Therapeutic Target of SAE and Sepsis-Associated Chronic Pain

Microglial activation facilitates brain damage during sepsis; thus, the modulation of microglial activation seems to be a relevant approach for treating SAE and chronic pain. For example, recombinant IL-17A can enhance neuroinflammation and microglial activation in caecal ligation and puncture mice. As expected, neutralizing antibodies against IL-17A or IL-17R can reduce CNS inflammation and microglial activation, thereby reducing cognitive dysfunction (Ye et al., 2019). Plus, several plant-derived substances, such as resveratrol, sophoraflavanone G (SG), attractylone, and β-elemene, have also been shown to inhibit microglial-mediated neuroinflammation and improve memory performance in mice with SAE (Guo et al., 2016; Sui et al., 2016; Pan et al., 2019; Tian et al., 2019).

In addition, studies of the roles of microglia in chronic pain have provided several new therapeutic approaches. And many efforts to inhibit microglial activation significantly reduced chronic pain. The strategies targeting on microglia can be roughly divided into three aspects. First, treatments targeting specific receptors necessary for microglial activation, such as TLR4, which has been reported to be a trigger of microglial activation (Bruno et al., 2018), the p38 MAPK pathways, and P2X4R. Second, treatments suppressing the release of microglial mediators, such as pro-inflammatory factors. Third, treatment targeting the mediators released by microglial. For example, low-dose naltrexone was shown to inhibit microglia activation similar to TLR4 inhibitor, and be an effective treatment for chronic pain in a pilot clinical trial (Younger and Mackey, 2009; Wang et al., 2016).

However, there are still many difficulties regarding robust and consistent laboratory results and clinical trials. One of the reasons is likely that microglia can also exert protective effects in the CNS. While cerebral synaptic activity is decreased in SAE, activated microglia insert their processes into the synaptic cleft to displace the axosomatic inhibitory synapses of neurons and thus increase neuronal excitatory activity, which results in the activation of synaptic NMDA receptors to exert anti-apoptotic and neuroprotective effect (Chen and Trapp, 2016). What’s more, complex interactions of microglia and other cells *in vivo*, polyphyletic effects of microglia-targeted drugs, differences between human and murine microglia, and distinct roles of microglia between female and male all can contribute to the disappointing clinical results. Thus, Ru-Rong Ji et al. proposed that the best strategy is to restore microglia normal function by pro-resolution approaches, such as pharmacological approaches, including CB2 (cannabinoid type 2) agonists, cell therapies and neuromodulation (Ji et al., 2016).

The endocannabinoid system (ECS) and its endogenous ligands play an important role on modulating the activation of microglia. The ECS mainly includes cannabinoid receptor type 1 (CB1R) and type 2 (CB2R), their small, lipid ligands (eCBs), and enzymes acting on synthesizing and degrading eCBs (Lutz et al., 2015). CB1R is mainly expressed in neurons, while CB2R is enriched in microglia (Stella, 2009). In neuroinflammatory and neurodegenerative disease conditions, CB2R is upregulated significantly in microglia, promoting phenotype alternation and anti-proinflammatory signaling in microglia (Tanaka et al., 2020). And some researchers have revealed that several molecules focusing on modulating CBR, such as endogenous fatty acid amide palmitoylethanolamide (PEA), can increase phagocytosis and migratory activity of microglia (Guida et al., 2017). Yet, further study is needed in designing microglial-targeted drugs that are effective in sepsis-associated chronic pain and in humans.

## Conclusion

SAE and chronic pain are frequent but severe complications of the dysregulated host response in sepsis. Numerous mechanisms have been implicated in the development of these conditions, among which activated microglia play a significant role. However, no specific treatments or standardized management suggestions are available in the current medical guidelines for sepsis and the associated complications. Therefore, the modulation of microglial activity, although still in the preclinical phase, may be an interesting therapeutic strategy.

## Author Contributions

All authors listed have made a substantial, direct, and intellectual contribution to the work and approved it for publication.

## Funding

This study was supported by National Nature Science Foundation Grant No. 81870961, the National Natural Science Shaanxi Province Natural Science Basic Research Programme-Key Projects (No. 2018JZ8004), and the Shaanxi Province Key Research and Development Programme (No. 2020ZDXM-SF-002) to XZ; the National Nature Science Foundation Grant No. 81801308 to ZF.

## Conflict of Interest

The authors declare that the research was conducted in the absence of any commercial or financial relationships that could be construed as a potential conflict of interest.
